# Eating practices during pregnancy: perceptions of select Maasai women in Northern Tanzania

**DOI:** 10.1186/s41256-017-0028-9

**Published:** 2017-03-13

**Authors:** Jessica Lennox, Pammla Petrucka, Sandra Bassendowski

**Affiliations:** 1 Graduate Student, College of Nursing, University of Saskatchewan, Saskatoon, Canada; 2 College of Nursing, University of Saskatchewan, Saskatoon, Canada

**Keywords:** Prenatal care, Maternal nutrition, Maasai, Tanzania, Interpretive description, Pregnancy

## Abstract

**Background:**

Globally, pregnant women are challenged to meet sufficient and necessary dietary intake in order to improve maternal and neonatal outcomes. These challenges are amplified in traditional communities, such as the Maasai, where the historical and cultural practices may further curtail, or impact on this dyad’s potential success. The research is intended to enhance understanding of Maasai women’s pregnancy and nutrition traditions as well as their beliefs.

**Method:**

Interviews with 12 pregnant Maasai women, all originally from the (Ngorongoro Conservation Area Authority NCAA) area and have spent most or all of their adult lives in the NCAA, sought to answer two research questions: how do these women describe their current dietary pattern and what do they believe is the role of nutrition during pregnancy.

**Results:**

Interpretive description methodology was used to reveal five themes: (1) Eating less food makes baby come easier, (2) Not producing food means more dependence, (3) Working hard harms my baby, (4) Knowing what is needed for a good pregnancy and (5) Preferring our traditional ways for pregnancy and birth.

**Conclusions:**

There is an imperative to address nutrition throughout the perinatal period within the Maasai population and the women recognize how important nutrition is for them and their babies. Opportunities to incorporate cultural values and practices must be embedded in programmes/services to achieve success and sustainability. It is important for future prenatal programming with the Maasai in northern Tanzania and other vulnerable groups of pregnant women to build on the women’s knowledge of what leads to good pregnancy outcomes.

## Background

Malnutrition is a leading cause of maternal and fetal complications in developing countries. Though food insecurity is the predominant cause of malnutrition, traditions and cultural beliefs surrounding nutritional practices during pregnancy can impact the nutritional status and outcomes. Acknowledging these cultural beliefs and traditions is an important global health consideration when endeavoring to improve maternal and child outcomes.

A number of studies considered traditional prenatal practices highlighting both diversities and commonalities. Wulandari and Whelan [1] state there is a wide range of ‘should and should not be eaten’ lists for pregnancy which are Indigenous-informed. Food avoidance with Ghanian pregnant women found a range from of avoiding meat, snails or certain vegetables to avoid a drooling or a ‘spirited’ child [2]. A study conducted in southern Tanzania revealed that 69% of the women avoided fish and farm meats [3]. Foregoing of eggs in parts of Tanzania and throughout parts of Africa is to assuage fears related to the animal’s characteristics being transferred to the child or sterility [4].

Some authors spoke hot/cold foods [1] and herbal remedies [5]. Many culturally informed prenatal food restrictions were related to ensuring that the ‘baby will not be too big’, head would be normal size, or to avert ‘a difficult labour’ [1, 4, 5].

Dietary taboos were most often enforced by the elders [2, 6]; mother-in-laws [4]; or husbands and other family members [7]. Oni and Tukur [8] found adherence to cultural practices tended to be more consistent in youth (teenage) pregnancies and less educated women, as well as in women with a low body mass index (a finding mirrored by Yassin, Sobhy, and Ebrahim [9]).

When the information from health providers differed from traditional practices, most women choose to follow cultural practices [2]. In a study by Mothupi [5], 12% of prenatal women in Nairobi, Kenya revealed their use of traditional practices such as herbal medicines often without the knowledge of the formal health care practitioners. In contrast a study in Zanzibar found women reported a fear of traditional medicine during pregnancy [10].

Within Tanzania, specifically, food insecurity remains the primary cause of under-nutrition and under-nutrition related illnesses. The Tanzanian Household Budget Survey (2011/2012) revealed that 28.2% of Tanzanians fell below the poverty line, an indication of the minimum consumptions of goods necessary for long-term physical wellbeing [11]. Additionally, 9.7% also fell below the food poverty line, classified as extremely poor and unable to meet the basic food needs of a household [11].

A study by Kalinjuma, Mafuru, Nyoni, and Modaha [12] assessed nutritional status of women using BMI, workload of women, birth weight, and current breastfeeding practices in four regions of Tanzania (i.e., Dodoma, Iringa, Njombe, & Singida). All women participants reported being responsible for cooking, washing clothes, and fetching water, with 60% indicating roles in care of children, taking care of invalids in the household, and collecting firewood [12]. The average birth weight for all regions was 3.24 kg, with 20% of births classified as LBW [12].

As previously stated, maternal weight prior to conception is a major determinant of LBW in infants, with maternal undernourishment during fetal development increasing the risk for developing macronutrient and micronutrient deficiencies during childhood [13]. The Tanzanian National Food and Nutrition Policy focuses on four major nutritional deficiencies affecting the population of Tanzania which include; protein energy malnutrition, nutritional anemia, iodine deficiency disorders (IDD), and vitamin A deficiencies [13]. The 2010 Tanzania Demographic and Health Survey (TDHS) showed 25% of children under age five were stunted and 17% were classified as severely stunted [13]. The TDHS revealed that 12% of children under age five are underweight (too thin for age) and 4% are wasted (too thin for height) [13].

Micronutrient deficiencies are prevalent in Tanzania, particularly iron deficiency anemia [14] and create significant vulnerabilities for the mother-child dyad [15]. According to the TDHS, 40% of women aged 15–49 were classified as anemic, a slight decrease from 48% in the 2004/2005 survey [13, 15].

The lack of prenatal education on nutrition and health during pregnancy, as well as accessibility to hospitals and clinics are other barriers to healthy maternal and child outcomes. Mosha and Philemon [16] reported factors influencing pregnancy outcomes in the Morogoro District of Tanzania with nearly two-thirds of the women knowing the right foods to eat during pregnancy but only 1 in three classifying fruits and vegetables as contributing to their iron status. Additionally, 63.7% of the participants listed meat, beans, lentils, and whole cereals as important foods to improve their general health [16]. A minority (3.2%) of women were unaware of the role of diet and nutrition throughout their pregnancy [16].

The Maasai rely on their herds of cattle, goats, and sheep as primary sources of income by selling or trading the meat and milk [17]. Traditionally, the diet was primarily meat, milk, and blood from domesticated animals. Due to land and grazing constraints, some Maasai, living outside of the NCAA, have begun to cultivate maize, rice, potatoes and cabbage to meet their nutritional needs [17].

To address diminishing food availability and decreasing cattle numbers in NCAA, the government of Tanzania has recently begun supplying free food rations and permitting Maasai to graze their cattle within the Ngorongoro Crater on the condition of daily entry and exit [18]. In October 2013, 7,000 tonnes of maize were delivered to the 87,000 NCAA residents with a commitment of annual ongoing support of 10 bags per family [19]. These foods are non-traditional to the Maasai, and, although they are addressing hunger related issues, they are not providing sufficient micro-nutrients and the implications to cultural nutritional practices are, as of yet, unknown.

In this context of change, challenge, and cultural variance in nutritional patterns, one must consider the impacts and implications of nutrition to the Maasai. Traditionally, Maasai women consume a modified diet, restricting caloric consumption during the third trimester, reducing intake of protein rich foods, and increasing water intake [20, 21]. Community elders often enforce this practice in the first pregnancy, although women, according to a number of the participants, may opt to follow this pattern in subsequent pregnancies. Powell [22] interviewed NCAA Maasai regarding their perceptions of dietary restrictions during the third trimester and found that the women viewed these nutritional restrictions as necessary for a safe delivery and to limit adverse medical outcomes. The women felt the dietary restrictions keep their bodies ‘clean’ during pregnancy in order to readily absorb nutrients contained in the perinatal diet [22]. According to Mawani [23], Maasai women in Kenya believed that it is important to continue a regular diet throughout pregnancy. Mawani’s study, however, noted that foods such as sugar, certain herbs, mutton, sheep’s offals and meat or milk from sick animals are avoided due to their perceived negative effects on the fetus [23]. These findings predate the food security interventions in NCAA and need to be revisited.

Maasai pregnancies and neonatal outcome are concerning. On average, Maasai women are observed to gain only 11% of their body weight during pregnancy [20] compared to their American and European cohorts with 15 to 25% gain [20, 24]. Approximately 13% of Maasai infants are categorized as low birth weight compared with an average 6.9% in the Organization for Economic Co-operation and Development countries [23, 24]. Many factors influence pregnancy outcomes, such as poor maternal nutrition, birth spacing, maternal age (under 15 years or over 35 years), inadequate prenatal care, lifestyle behaviors, and poverty [22–24]. It is in this context of the changing food availability and food insecurity affecting the maternal-child dyadthat we framed our study.

## Method

This qualitative descriptive study explored the views and daily dietary habits of select pregnant Maasai women currently or previously from the NCAA. Using an interpretive description methodology, which is rooted within a phenomenological qualitative approach, the researchers sought to answer two research questions: 1) What are the beliefs of pregnant Maasai women on the roles of nutrition in healthy pregnancy outcomes? and 2) How do pregnant Maasai women from the NCAA describe their current dietary pattern? Data was collected in two optional parts: a semi-structured individual interview (see Appendix) lasting between 15 and 60 min; and/or a 24-h diet recall conducted immediately after the interview which took place in November 2015. However, the research team has an ongoing relationship in this community for more than 4 years. Each woman was invited and chose to complete both parts, but was given the option to participate in either or both. This article considers the interview (results) contributions and perspectives of the participants respecting the first research question. The dietary recall analysis and second research question will be published in a subsequent document.

A convenience sampling method yielded 12 study participants through a combination of posters and ‘word of mouth’ recruitment. Posters translated in to Swahili and M’aa were placed in local clinics and the ward administrator’s office in the village. Due to literacy and logistical issues (travel to the village) efforts were made to ensure the largest number of women were made aware of the research opportunity through word of mouth. The local clinic staff and administration were important in letting members of the community know about the opportunity and asking them to share it with any woman who might be interested and appropriate for the study purposes. The two interviews in the city locale were through word of mouth (telephone contacts from members of the community to the women about the project).

The project team had intended to interview between 6 and 8 individuals; however, the successful recruitment was seen as an opportunity to include more voices and perspectives. All women were originally and/or currently from the NCCA, although 10 interviews occurred in Meshili village and 2 in Arusha city. To be eligible to participate, women must have self-identified as Maasai woman in any trimester of pregnancy. All women provided voluntary informed consent, either verbally or written, depending on their literacy level. Interviews were conducted as one to one interactions lasting about 20 to 60 min each by either the research assistant or the primary author. The NCCA based interviews all took place at either the local village dispensary or at the woman’s home based on the participant’s request. The Arusha based interviews took place at the offices of a local partner non-government organization. In each case the setting provided privacy and ensured confidentiality.

Demographic data was recorded prior to each interview. An interview guide was created by the first author with translation done first to Swahili then to M’aa as many terms and phrases are existing in the former language but have been adapted into M’aa. Translation was done by 2 Swahili native speakers independently into Swahili and consensus was reached before the research assistant translated the content to M’aa. The village based interviews were conducted by a male tri-lingual research assistant who works as a community health service provider in the community. All village-based interviews were conducted in M’aa and translated to English. In Arusha the interviews were conducted by the primary author with the research assistant in attendance to assist in case language became an issue. Each interview was digitally recorded, translated, transcribed, and edited with the primary author and local research assistant, with a special focus on clarifying any culturally specific words, concepts, and/or practices.

Interpretive description was selected as the qualitative methodological approach to distinguish commonalities between what was already known regarding beliefs of pregnant Maasai women and the findings of this study, allowing interpretation of new data and application of evidence to practice. This non-categorical research methodology encompasses multiple qualitative methods to describe the complex interactions between psychosocial and biological phenomena [25]. The study used interpretive description in order to generate new constructs out of the data which forces the researchers to “see beyond the obvious, rigorously testing out that which we think we see, and taking some ownership over the potential meaning and impact of the visions” [26].

Thorne et al. [26] advises researchers to keep data analysis simple and to avoid removing the researcher from the participants, which could harbor over analyzing and misinterpretation of participants’ views. All the data were analyzed using content analysis, a technique for creating replicable interpretations from qualitative data such as interviews, observations, and other contextually important materials [27]. The purpose of content analysis is to arrange large amounts of text into themes and subthemes with similar meaning and generate a deeper interpretation through coding and identifying commonalities and themes within the findings [28].

### Analysis

The interview results were analyzed using Excel™ software, by highlighting or coding common words, phrases, and subjects. To further reflect the participants’ voices, in most cases, the theme label (name) was derived from an actual participant quotation. The final 5 themes were reviewed and agreed to by the research team and the research assistant.

## Results

Participant ages ranged from 18–30 years (please note that actual age is not always easily determined as birth records are often not kept). One participant was primigravida (8.3%) with the remainder multigravida (91.6%) with most reporting 2 or 3 children. All (100%) participating women were married. All participants were originally from Olbalbal communities. At the time of the study, a majority (83.3%) of participants lived in the rural district of Olbalbal in the NCAA. One (8.35%) participant was currently living in Arusha City with her husband and the other (8.35%) was doing business in the city on an intermittent basis (going back to Olbalbal regularly).

As mentioned the study had two major components – an individual interview and a food recall. These results reflect the former, whilst the food recall will be reported in a second paper.

Through data analysis, five common themes were identified:

- Eating less food lets baby come easier;
- Not producing food means more dependence;
- Working hard harms my baby;
- Knowing what is needed for a good pregnancy; and
- Preferring our traditional ways for pregnancy and birth.

It is noted that the limited number of primigravidas precluded the discussion of results based on number of pregnancies.

### Theme 1: ‘Eating less food lets baby come easier’

This theme, which derives from a direct quotation, reflects the belief that a pregnant woman’s food intake must be decreased to prevent a large baby. This belief seemed to relate to a number of factors such as risk of death due to a large infant, concern for lack of specialized care if the baby did not pass naturally, and concern over health of a big infant.

A majority of the women travel long distances to clinics and value the tradition of giving birth at home under the supervision of a traditional birth attendant. Delivering at home makes it necessary for women to avoid big babies, which, they believe, increases the risk for a cesarean delivery. One woman stated *“If I eat meat it will make the baby fat that would make me go to the hospital and deliver through surgery.”* (Participant 9 [P9]).

The women told of the Maasai traditions surrounding food during pregnancy which restricted or prevented them from consuming unpasteurized milk, meat, or milk from cattle (other than their own), eggs, sweet foods, and butter. Also, they shared that women were to restrict caloric intake, especially from sweet or fatty foods throughout their pregnancies. When asked “What foods do you avoid while you are pregnant?” one women replied *“beans and milk, because if you eat this, the baby will be fat”* [P6]. These traditions restrict women from eating any meat or drinking milk from their sixth month of pregnancy until delivery. At delivery, they are expected to be “clean” in order to readily absorb nutrients contained in the postnatal diet.

*… any sweet foods aren’t allowed, I am not allowed to eat the fat separated from the milk (butter) until I am closer to birth… We avoid those foods because we don’t want the baby to get really really fat..*. [P2]

In combination with the dietary restrictions, many pregnant Maasai women reported a decrease in appetite during their pregnancy, which, when combined with nausea and vomiting, resulted in further restriction in dietary intake. All participants interviewed reported feeling very tired since becoming pregnant. One woman said, *“Sometimes I wake up feeling very tired and nauseous…I feel drunk but haven’t had anything to drink…when I feel that way I just drink water or anything sour.”* [P2]

According to the participants, in a Maasai household, the male head is always the first to eat followed by the children, and lastly, the mother. Most participants interviewed reported being fed last as having no effect on how much food they received. However, according to Participant 2:

*Since I’ve been pregnant and am told not to eat certain foods, they don’t see me as a priority to have to eat first. Sometimes I’m not full but I’m supposed to stop eating (be)cause I’ve reached my limit…and sometimes if we run out of food I have to drink tea with milk instead. There’s nothing that I can do because it’s the tradition*.

The use of local herbs and medicine at some point during a pregnancy is seen as a means to cleanse or cure the woman of sickness (i.e., nausea and vomiting), fever, or from consumption of ‘prohibited foods’ (i.e., foods high in fat or sugar). On average, the Maasai women interviewed were given local medicine consisting of naturally grown herbs once a week to induce vomiting and diarrhea. Primarily, the mother-in-law or Elders monitor the pregnant mothers’ diets. One participant [P11] reported that *“[I] take these medicines to have an appetite to eat more…to feel light”.* One of the two women currently located in the city discussed how Maasai cultures and traditions differ when they live in the NCAA.

*I am a Maasai that lives in town so there’s no one to restrict my diet, so sometimes I eat whatever I want but sometimes if I eat something that I don’t think is healthy I’ll take the medicine myself to make myself vomit.*

Consistent with Maasai traditions, study participants believe reducing dietary intake will avert ‘big’ babies and complications during childbirth. Pregnant mothers rely on the experience of relatives and Elders in the community to guide them through pregnancy and delivery without formal health system interventions.

### Theme 2: ‘Not producing food means more dependence’

Maasai historically relied on cattle, sheep, and goats as their principal dietary sources. As herd sizes have decreased the Maasai increasingly rely on food brought in to the NCAA on market days and relief foods. There is an increasing dependence on non-traditional foodstuffs, a situation which the women struggle with. On bi-monthly market days, women walk long distances to the village to purchase fruits and vegetables, but not all women who attend the market are successful in making purchases due to cost and supply. When asked about their food sources, six women reported purchasing maize, seven indicated getting milk from their cows, and three obtained vegetables from the forest.

Very few Maasai families access food outside of NCAA due to lack of transportation and funds, limiting their diets to milk, maize, and meat derived from cows, sheep, or goats. This situation has created an inability for self-sustainability and a greater dependency on purchased or government relief foods. When asked to explain Maasai traditions surrounding diet during pregnancy, one woman explained:

*Mostly what [Maasai women] eat in Ngorongoro is maize, maize flour, milk, and meat/intestines. The goat meat is from our own herd of goat, we never take meat or livestock from anywhere else because we don’t know if they were on any medication or sick.* [P2]

In the NCAA, seasonal changes affect food availability, altering which foods are naturally available as those brought in for purchase. During the rainy season, most (7) women reported eating vegetables. One woman indicated that *“During the rainy season I get vegetables and ugali (stiff porridge), and during the dry season I get only porridge.”* [P8] Only three women reported eating any form of protein or dairy product during the dry season, with a majority reporting eating only maize, and maize porridge. As mentioned previously these latter foods, which lack the requisite micronutrients, often comprise the bulk of the government relief foods.

### Theme 3: ‘Working hard harms my baby’

Women in the community are responsible for household chores, such as collecting firewood and water, taking care of children, and food preparation. Pregnant Maasi women will steadily increase their workload throughout the second and third trimesters in preparation for the postnatal period, when they will remain in their bomas (homes) for three months post-partum to recover from childbirth and to care for their newborns [14].

Many women described feeling hungry, tired, and weak throughout their pregnancies. The increased workload and decreased nutritional support compromises the women’s abilities to maintain the energy level necessary to perform activities of daily living. Half of the women interviewed described difficult environments and increased workload as being detrimental to a healthy pregnancy. Participant 2 explained that walking long distances while pregnant became increasingly difficult saying, *“they don’t care if I’m pregnant or not I still have to do my daily chores like, walking to get water and firewood which usually involves long distances”*.

Daily chores become increasingly difficult to perform in the heat and there is little protection from the environment. Some women interviewed indicated a choice in sacrificing clinic visits and checkups to avoid walking long distances in these extreme conditions. One woman stated

*What I am going through I don’t feel anything good at the moment, the only thing good about the pregnancy is about having the baby.* [P2]

### Theme 4: Knowing what is needed for a good pregnancy

Many participants shared instinctual knowledge on how to maintain healthy pregnancies, even though such knowledge may contradict cultural beliefs. Cultural pregnancy beliefs and traditions enforce rules or guidelines regarding diet, activity level, and rest. Many of those interviewed understood that not all traditions are beneficial. Most participants had a basic understanding of good nutrition during pregnancy, with eight indicating that they had adhered to this healthy, traditionally ascribed approach throughout their pregnancies.

With respect to local medicines and herbs, the women indicated usages such as “*to clean out”, “to help cooling down our bodies”,* and *“for fever”.* Rather than labeling traditional medicines as good or bad, they described them as necessary to ensure health and to address over indulgences. In discussion of what was good and bad during their pregnancy, six women mentioned hunger and lack of food as being bad during pregnancy, as epitomized by one woman stating that *“Good, if I get enough food, and bad, if I am hungry”* [P5]. The other half of participants indicated that not enough rest, and difficult work are bad for their pregnancies. In addition, some mentioned anger as being harmful during pregnancy. One woman stated,

*I wish I could change how much anger I have, (be)cause in Maasai traditions you are not supposed to be an angry person during pregnancy…it is [not] healthy for my baby to be mad all the time.* [P1]

Lastly, the women were asked if there was anything that they could or should change to help the baby’s development during pregnancy. Three women voiced *“having resting time”* [P6] as something they could change to help baby develop healthy and safely. Three women said having a balanced diet was important “*to try to find balanced diet which my baby loves.”* [P11]

### Theme 5: Preferring our traditional ways for pregnancy and birth

Maasai cultures and traditions impact on prenatal and postnatal care, both positively and negatively. This life stage, according to the participants, is filled with *“family”*, including mothers-in-laws, husbands, parents, and other wives in their polygamous relationships. One woman stated, *“For us we get help from women Elders who have already gave birth and are years older…I need to be close to someone that has experience in the community.”* [P2] The Elders are partially responsible for monitoring food intake, and administering local medicines. Family members, community members, ‘Wakunga’ (local traditional birth attendants), and Elders share responsibility for monitoring food intake during pregnancy. One woman reported:

*The mother in law monitors my diet, if she sees me eating foods that she believes are bad for baby, too fatty, or will make baby over weight, she will make me drink a special drink to induce vomiting…given on average once a week…women have to hide while eating when they are pregnant so the elders don’t see them eating so much.* [P2]

Nine of twelve women reported taking some form of local medicine during their pregnancies. One woman reported *“I take local herbs “Oloiren” and “Oloisuki”* [P7]. Herbs are taken during times of sickness or after eating fatty foods as a way to “clean out” the mother. Another discussed *“there is a medicine we call black okeshal [or] “Olevisi”, it is used for pregnant women to help with cooling down our bodies, and helps with hydration”* [P2].

## Discussion

There is minimal research evidence on how Maasai women’s perception of the importance of diet and nutrition during pregnancy and to healthy child outcomes. Although cultural traditions dictate the diet and activity levels, many participants understood the importance of a balanced diet and adequate rest during pregnancy. The women indicated a preference for adhering to traditional practices during pregnancy as they were supported by other women and being cared for in a traditional way (by elders and traditional birth attendants). So there is a dissonance between knowledge of dietary needs during pregnancy and adherence to follow cultural practices.

The study suggested the imperative for promotion of prenatal care to invite women to openly discuss traditional and mainstream practice in order to have healthier pregnancies and healthier babies. The women talked about feeling tired and overworked, not getting enough to eat and lacking vegetables during their pregnancy, indicating an awareness of how these contribute to an unhealthy pregnancy. They also shared their choices to have healthier babies such as eating less to ensure the baby could ‘pass’, avoiding certain foods that are culturally regarded as bad for the baby, and taking local herbs.

This is a critical opportunity for knowledge mobilization, with the research evidence from this study, to bring forward new ideas, options, and an informed dialogue on prenatal nutrition concerns and needs amongst Maasai women. Such a dialogue must be culturally respective and inclusive.

This study also showed the interest and willingness of this group of women, to share their experiences in the hope of improving their situation and that of other pregnant women.

Opportunities for ongoing research and knowledge translation should be considered, which are inclusive of the elders, traditional birth attendants, local health providers, and the pregnant women.

There were a number of limitations in this study. The first limitation relates to development of the interview guide. As there was no specific pre-existing tool so an interview guide was developed. Although the instrument used open ended questions, most women often answered with only one to two word responses. Future iterations might include more extensive questioning to enable multiple ways of seeking answers or conduct group interviews which might invite more extensive dialogues. The perceived position of power of the interviewer/researcher may have contributed to this succinct answering pattern. To address this, one might consider a more prolonged engagement in the community which potentially generates familiarity and comfort with process and person. A second limitation was reliance on a local research assistant to collect the interviews and dietary recalls. Although the research assistant was a local community member of Maasai descent, and fluent in M’aa, Swahili, and English, he was male. This interface could have created bias and/or limited the interview content by creating a perceived power imbalance in the interviewer/interviewee relationship or making the female participants uncomfortable to discuss their health and pregnancy with a male. This gender difference may have also yielded under-reporting by the women due to perceived ‘traditional’ roles and expectations. A third limitation was in the need for multiple translations. Nes, Abma, Jonsson, and Deeg [29] discussed how language difference in qualitative research may have consequences in loss of meaning, or the misinterpretation of words or how they are perceived. In this study, complexity was added in needing a translator who was trilingual. In order to ensure reliability of interpretation throughout the data transcription process, the research assistant and lead researcher worked together to clarify each relevant word and interpretation of the participants’ meaning to ensure authenticity of each interview. Some risks associated with translation were potentially mitigated through digitally recording interviews which provided the opportunity to cross-check certain words or phrases with the translator and the local doctor (both of whom were tri-lingual), helping with the validation of findings as described by Murray & Wynne [30].

## Conclusion

This research described traditional and current dietary practices of select Maasai women during pregnancy and their perceptions on how diet and nutrition impact maternal and child outcomes. The women interviewed variably described restricted dietary intake throughout their pregnancies, especially during the third trimester, with the intent of reducing the size of the baby to facilitate easier delivery. Added chores, excessive walking, and difficult workloads during pregnancy further increases metabolic expenditure, further reducing fetal size.

The study findings may contribute to improving nutritional status of pregnant Maasai women by providing knowledge of cultural practices related to pregnancy nutrition. Community leaders, family members, and traditional birth attendants are influential in how and when the Maasai women seek medical care throughout their pregnancy.

From the research findings, gender inequality in relation to inadequate nutritional patterns, and prenatal workload informs potential research direction. Locally, this research may inform program changes by embedding traditional beliefs, focus on nutrition, and on evidence based practices. By promoting an understanding of the mechanisms and risks of fetal growth restriction, cultural practices may change and encourage the reconsideration of food restrictions in pregnant Maasai women.

The research findings can as well be used as a catalyst in development of local policies by focusing on accommodating traditional beliefs respecting culturally safe prenatal care and nutrition. These research findings can potentially inform government policies specifically in the domain of food aid programs, by increasing a focus on variability of relief foods with the imperative for more nutrient dense foods, specifically for pregnant and lactating women.

## Appendix

### Interview guide

What types of food do you eat most day?

How do your foods differ during rainy or dry season?

Where do you get your food from?

Would you say you eat more/less/about the same amount now that you are pregnant?

How much more do you eat now that you are pregnant?

In your household, who is fed first? Who is fed last? How does this affect how much food you get? How does this affect what food you get?

How have you been feeling so far during your pregnancy? Strong? Tired? Upset stomach?

Do you feel sick, or do you vomit during your pregnancy? Do you take local herbs or local medicine to help with sickness? If so, what kinds of herbs?

Can you explain what Maasai traditions for diet/foods you follow during pregnancy?

What traditional foods do you eat while you are pregnant? Why?

What foods do you avoid while you are pregnant? Why?

What do you think helps make the baby healthy while you are pregnant? Do you do this in your own pregnancy?

Can you tell me about your pregnancy so far?

Tell me about what things or people help you during your pregnancy? e.g., family members, friends, local health providers (traditional birth attendants).

Tell me about anything that makes it hard to have a healthy pregnancy/ healthy child?

Tell me what you know about what is good for you during pregnancy and what is bad?

Is there anything you feel that you can or should change to help your baby grow during your pregnancy?

Is there anything you want to ask me?

## Abbreviation

NCAA: Ngorongoro conservation area authority
